# Editorial: Rising stars in virology: 2022

**DOI:** 10.3389/fmicb.2023.1205877

**Published:** 2023-05-30

**Authors:** Yasuko Tsunetsugu-Yokota, Gkikas Magiorkinis, Hirokazu Kimura

**Affiliations:** ^1^Department of Human Sciences, Tokyo University of Technology, Tokyo, Japan; ^2^Department of Hygiene, Epidemiology and Medical Statistics, Medical School, National and Kapodistrian University of Athens, Athens, Greece; ^3^Department of Health Science, Gunma Paz University Graduate School, Takasaki, Japan

**Keywords:** HDV, cat hepadnavirus, Ebola virus, SFTS virus, epidemiology, novel technology

In this Research Topic, we collected articles submitted by five early-stage investigators who have fresh ideas and are going to develop their careers. The subjects include a wide variety of viruses, concerning epidemiology (Chang et al.; Shofa et al.), and technology development (Furuyama et al.; Suther et al.; Zhu et al.).

Chang et al. assessed more than 1 million blood donors in a multi-center epidemiological study in China and showed a low anti-HDV seroprevalence among HBVAg+ blood donors, thus suggesting a low risk of HDV transmission via blood transfusion. The review article by Shofa et al. summarized the current knowledge of cat hepadnavirus on epidemiology, host tropism, and potential pathogenicity. This virus was recently discovered in Australia and has been recognized worldwide. As a novel tool, Furuyama et al. developed a system of tetracistronic transcriptional virus-like particles (trVLP) possessing fluorescent glycoprotein (GP) for the visualization of the virus life cycle. Because their trVLP system is replication-competent, GP-mediated entry and GP transport of the Ebola virus can be directly followed *in vitro*, which is suitable for drug screenings in BSL-2 conditions. Also, Zhu et al. utilized the CRISPR-Cas technology and developed a system that allowed the simultaneous detection of two genes in the genome of the severe fever thrombocytopenia syndrome (SFTS) virus. The system will be practically useful for the on-site detection of the SFTS virus. Lastly, Suther et al. reviewed the reported performances of isothermal amplification methods for detecting foodborne and enteric viruses, methods that have been shown to have very good performance in terms of portability, diagnostic accuracy, turnaround time, and minimal workload and lab process. The authors compared those methods focusing on the sensitivity and treatment of food environmental test samples.

Once again the Research Topic “*Rising Stars in Virology*” demonstrates the innovation and wide range of fresh ideas by early stage researchers, that should be encouraged and supported. It is evident from the 1,327 downloads and 6,599 downloads of the articles in this Research Topic, that the rising stars will make an impact in the field of virology.

## Author contributions

YT-Y wrote the original editorial and GM edited it. All authors contributed to the article and approved the submitted version.

